# Prevalence and Distribution of Antimicrobial Resistance-Associated Mutations in *Mycoplasma genitalium* Identified Through Routine Molecular Diagnostics in Korea

**DOI:** 10.3390/microorganisms14030665

**Published:** 2026-03-15

**Authors:** Ho-Jae Lim, Yoon-Taek Hong, Seung-Hui Baek, Min-Young Park, Min-Jin Kim, Yong-Hak Sohn, Yong-Jin Yang

**Affiliations:** Department of Molecular Diagnostics, Seegene Medical Foundation, Seoul 02637, Republic of Korea; 52rotc.hjl@mf.seegene.com (H.-J.L.); koreahyt@mf.seegene.com (Y.-T.H.); sh1113@mf.seegene.com (S.-H.B.); pyli186@mf.seegene.com (M.-Y.P.); lithium2864@mf.seegene.com (M.-J.K.); medsohn@mf.seegene.com (Y.-H.S.)

**Keywords:** *Mycoplasma genitalium*, multiplex polymerase chain reaction, resistance-associated mutations, macrolide, fluoroquinolone, molecular surveillance

## Abstract

*Mycoplasma genitalium* is a significant sexually transmitted pathogen, and its clinical management is increasingly complicated by the global distribution of mutations associated with macrolide and fluoroquinolone resistance. To characterize the molecular resistance landscape in a routine diagnostic setting, we retrospectively analyzed residual clinical specimens collected during routine sexually transmitted infection testing between January and December 2024. Among 374,021 specimens screened, we included 4019 *M. genitalium*-positive samples containing sufficient residual material. Using multiplex polymerase chain reaction assays, we detected mutations associated with macrolide and fluoroquinolone resistance in the 23S rRNA and *parC* genes, respectively. Frequent substitutions included A2059G and A2058G in the 23S rRNA gene (1253 samples) and substitutions at positions 248 and 259 in the *parC* gene (1306 samples). Mutations in the predefined 23S rRNA and/or *parC* targets were identified in approximately 44% of the analyzed samples, with distinct patterns of mutation distribution and co-occurrence. Although phenotypic susceptibility and clinical outcomes were not assessed, this large-scale, assay-based analysis provides a comprehensive overview of resistance-associated mutation patterns in *M. genitalium* derived from routine diagnostics, supporting molecular surveillance for monitoring antimicrobial resistance trends.

## 1. Introduction

*Mycoplasma genitalium* is an established sexually transmitted pathogen associated with nongonococcal urethritis in men and cervicitis, pelvic inflammatory disease, and adverse reproductive outcomes in women [1]. Since its recognition as a human pathogen, *M. genitalium* has emerged as a clinically significant pathogen owing to its role in persistent and recurrent urogenital infections [2]. However, diagnosis and management remain challenging because of its distinct biological features, including a small genome (approximately 580 kb), slow growth, and the absence of a cell wall, which complicate laboratory detection and antimicrobial treatment [3,4,5].

A major challenge in the clinical management of *M. genitalium* infection is its fastidious nature, precluding reliable culture in routine clinical settings [6]. Consequently, traditional culture-based antimicrobial susceptibility testing is impractical, and laboratory diagnosis relies almost exclusively on nucleic acid amplification tests (NAATs) [7]. The widespread adoption of NAATs has substantially improved diagnostic sensitivity and enabled the direct detection of *M. genitalium* from clinical specimens [6]. Advances in molecular diagnostics have enabled the detection of genetic mutations conferring antimicrobial resistance, providing an alternative approach for monitoring resistance patterns in this organism [8].

Over the past decade, studies have reported increasing rates of antimicrobial resistance in *M. genitalium*, particularly to macrolides and fluoroquinolones, which are the primary and secondary treatment options in several clinical guidelines [9,10,11]. Macrolide resistance is most commonly associated with point mutations in the 23S rRNA gene, particularly at positions A2058 and A2059 (reported using *Escherichia coli* numbering), which reduce macrolide binding to the ribosomal target [4,12]. Fluoroquinolone resistance has been linked to mutations in the quinolone resistance-determining region of the *parC* gene, with substitutions at positions corresponding to amino acids S83 and D87 commonly reported [13]. These mutations reduce the effectiveness of standard treatment regimens and contribute to treatment failure and persistent infection [14].

Notably, the prevalence of macrolide- and fluoroquinolone-associated mutations in *M. genitalium* varies considerably across geographic regions, populations, and clinical settings [15]. Surveillance studies in Europe, Australia, and parts of Asia show significant regional differences in mutation prevalence, reflecting variation in antimicrobial prescribing practices, diagnostic strategies, and population-level antimicrobial exposure [13]. These findings highlight the need for locally relevant data to guide clinical decision-making and public health strategies [13,16].

Therefore, in response to the growing burden of antimicrobial resistance, international and national clinical guidelines increasingly recommend resistance-guided treatment strategies for *M. genitalium* [11,17,18,19,20]. For example, in Korea, the 2023 national guidelines recommended resistance-guided treatment approaches for *M. genitalium* [11], broadly consistent with guidelines from the United States and European [9,10] while reflecting local trends in antimicrobial resistance. These strategies involve the detection of resistance-associated genetic mutations before or at diagnosis, enabling clinicians to tailor antimicrobial therapy and avoid ineffective regimens [21]. However, the effective implementation of resistance-guided management requires timely, accurate, and population-specific data on the distribution of resistance-associated mutations [13,16], and in several regions, including Korea, comprehensive and current surveillance data from routine clinical practice remain limited [16,22].

Multiplex polymerase chain reaction (PCR) assays that simultaneously detect *M. genitalium* and key resistance-associated mutations have become practical tools to address this gap [23]. By targeting predefined regions within the 23S rRNA and *parC* genes, these assays enable the rapid, high-throughput identification of clinically relevant mutations directly from clinical specimens, without the need for culture [15,24]. The integration of these assays into routine diagnostic workflows facilitates the generation of large-scale surveillance data that reflect real-world clinical practice rather than selected research cohorts [21,25].

Beyond assessing the prevalence of individual resistance-associated mutations, it is crucial to evaluate mutation co-occurrence across antimicrobial classes is crucial [14,26]. Concurrent mutations in the 23S rRNA and *parC* genes confer reduced susceptibility to macrolides and fluoroquinolones, potentially limiting treatment options [14,26]. Studies have reported the emergence of these combined mutation patterns, underscoring the need for integrated molecular surveillance that accounts for both mutation prevalence and specific mutation combinations [14,26].

Furthermore, demographic and clinical factors, including sex and specimen type, may influence the observed distribution of resistance-associated mutations [13,14]. Differences in healthcare-seeking behavior, anatomical sampling sites, and prior antimicrobial exposure may contribute to the observed sex-associated variation in mutation prevalence [27,28]. Nevertheless, as evidence supporting these associations is lacking, studies involving large, representative datasets are needed to clarify these relationships [13].

In this study, we analyzed residual clinical specimens that were collected during routine sexually transmitted infection testing between January and December 2024 by the Seegene Medical Foundation, as previously described [29]. Using these specimens, we characterized the prevalence, distribution, and co-occurrence patterns of mutations associated with macrolide and fluoroquinolone resistance in *M. genitalium*. Resistance-associated mutations were detected using multiplex PCR assays targeting predefined regions of the 23S rRNA and *parC* genes. This approach enabled the generation of large-scale, assay-based surveillance data to be generated directly from routine diagnostics. By integrating mutation distribution analyses with demographic characteristics, including sex-associated patterns, this study provides a comprehensive overview of the molecular resistance landscape of *M. genitalium* in Korea.

## 2. Materials and Methods

### 2.1. Clinical Specimens

This study was approved by the Institutional Review Board of Seegene Medical Foundation (SMF-IRB-2024-003; 14 March 2024). The requirement for informed consent was waived because all samples collected for this study were anonymized.

We initially identified 374,021 clinical specimens from individuals who underwent routine diagnostic sexually transmitted infection testing between January and December 2024. Among these specimens, 9590 were positive for *M. genitalium*, as previously described [29]. Of the *M. genitalium*-positive samples, 41.9% (4019/9590) contained sufficient residual material to conduct resistance testing targeting macrolide- and fluoroquinolone-associated mutations and were included in the final analysis. Inclusion in the resistance analysis was determined solely by residual specimen availability and was not based on clinical characteristics or mutation status. Comparative demographic data between the analyzed samples and the full *M. genitalium*-positive cohort were not available.

Specimens were categorized in the laboratory information system as either urine or swab samples according to routine diagnostic workflows. Detailed anatomical site information (e.g., cervical, vaginal, urethral, or rectal) was not available in the anonymized dataset used for this analysis.

### 2.2. Sample Processing and Nucleic Acid Extraction

Urine samples (10 mL) were prepared following the manufacturer’s instructions. Swab specimens were preserved by swirling the swab in the transport medium, without additional pretreatment. We performed nucleic acid extraction (Maelstrom 9600 platform; Taiwan Advanced Nanotech Inc., Taoyuan, Taiwan) using STARMag^TM^ M96 kits (Seegene Inc., Seoul, Republic of Korea).

Briefly, 200 µL of each sample was transferred into a lysis buffer cartridge and loaded into the instrument with washing reagent cartridges, a pipette tip holder, and elution plates. Extraction was automated following the manufacturer’s protocol.

### 2.3. Detection of Resistance-Associated Mutations in M. genitalium

All PCR assays were performed on the CFX96 Real-Time PCR Detection System (Bio-Rad Laboratories, Inc., Irvine, CA, USA) following the manufacturer’s instructions. We used the Allplex^TM^ MG & MoxiR Assay (Seegene Inc.) to detect *M. genitalium* and *parC* mutations (A247C, G248A, G248T, G259A, G259C, and G259T) associated with reduced fluoroquinolone susceptibility [15,24,30].

We detected 23S rRNA gene mutations associated with reduced macrolide susceptibility (A2058C, A2058G, A2058T, A2059C, A2059G, and A2059T; *E. coli* numbering) using the Allplex™ MG & AziR Assay (Seegene Inc.) [15,24,31,32]. Amplification data were analyzed using Seegene Viewer software (version 3.33; Seegene Inc.). Both multiplex real-time PCR assays were performed under identical cycling conditions as follows: initial denaturation at 95 °C for 15 min, followed by 45 cycles of denaturation at 95 °C for 3 s, annealing at 60 °C for 10 s, and extension at 72 °C for 10 s. Data were analyzed using Seegene Viewer for Real-time Instruments (version 3.33; Seegene Inc.).

### 2.4. Statistical Analysis

Continuous variables are presented as median (interquartile range, IQR), and categorical variables are presented as counts and percentages. Differences in continuous variables between groups were evaluated using the Mann–Whitney U test, and categorical variables were compared using Pearson’s chi-square test without continuity correction. Statistical analyses were performed using R software (version 4.2.2; R Foundation for Statistical Computing, Vienna, Austria) for Windows. All tests were two-sided, and *p*-values < 0.05 were considered statistically significant.

## 3. Results

### 3.1. Study Sample Characteristics

As shown in Table 1, the overall median age was 30 years (interquartile range [IQR], 25–38). Male-associated samples were obtained from significantly older individuals than female-associated samples (32 vs. 28 years; Mann–Whitney U test, *p* < 0.001). Sample source differed significantly by sex (χ^2^ test, *p* < 0.001), with urine specimens predominantly obtained from male individuals (1868/1882, 99.3%) and swab specimens primarily obtained from female individuals (2082/2137, 97.4%).

### 3.2. Macrolide Resistance-Associated Mutations in the 23S rRNA Gene

Of the 4019 *M. genitalium*-positive samples analyzed, 1253 harbored 23S rRNA gene mutations associated with macrolide resistance. The distribution of individual mutations is summarized in Table 2. A2059G was the most common (52.8% of mutation-positive samples), followed by A2058G (34.7%). A2058C was uncommon (0.4%), and A2059T was not detected. Concurrent mutations at the A2058 and A2059 positions were identified in 10 samples. In addition, 34 samples had two substitutions at a single position. This included 33 samples with G and C substitutions at A2059 and one sample with G and T substitutions at A2058. Sex-stratified analysis showed that the proportional distribution of individual 23S rRNA mutation subtypes was generally comparable between male- and female-associated samples. Macrolide resistance-associated mutations primarily reflected recurrent substitutions within the analyzed regions of the 23S rRNA gene.

### 3.3. Fluoroquinolone Resistance-Associated Mutations in the parC Gene

Fluoroquinolone resistance-associated mutations in the *parC* gene were identified in 1306 samples (Table 3). G248T was the most common mutation (52.2%), followed by G259A (19.8%) and G248A (11.8%). Mutations G259C (1.1%), A247C (7.0%), and G259T (8.3%) were less frequently detected. One sample exhibited concurrent mutations at positions 247 and 248, and three samples harbored concurrent mutations at positions 248 and 259. The sex-stratified distributions presented in Table 3 demonstrate broadly similar patterns between male- and female-associated samples. Fluoroquinolone resistance-associated mutations reflected recurrent substitutions in the *parC* gene.

### 3.4. Combined Mutation Patterns and Sex-Associated Differences

To characterize the distribution of concurrent mutations, we jointly assessed mutations associated with macrolide resistance (23S rRNA gene) and fluoroquinolone resistance (*parC* gene). Based on this combined analysis, *M. genitalium*-positive samples were categorized according to the presence of mutations in the 23S rRNA gene only, the *parC* gene only, both genes, or neither of the two genes. The distribution of these combined mutation patterns is summarized in Table 4.

Mutations in the predefined 23S rRNA and/or *parC* targets were detected in approximately 44.3% of the analyzed *M. genitalium*-positive samples, of which 19.4% harbored concurrent mutations in both targets. Sex-stratified analysis revealed that the overall prevalence of resistance-associated mutations was significantly higher in male-associated samples than in female-associated samples (48.5% vs. 40.6%; χ^2^ test, *p* < 0.001). Similarly, 23S rRNA mutations and concurrent 23S rRNA and *parC* mutations were more common in male-associated samples (*p* = 0.003 and *p* < 0.001, respectively), whereas the prevalence of *parC* mutations did not differ by sex (*p* = 0.906).

## 4. Discussion

In this study, we present the results of a large-scale analysis of mutations associated with macrolide and fluoroquinolone resistance in *M. genitalium* detected through routine multiplex PCR diagnostics. By analyzing residual clinical specimens collected during routine sexually transmitted infection testing, we characterized the prevalence, distribution, and co-occurrence patterns of resistance-associated mutations in a real-world setting.

A key finding of this study is the high overall prevalence of mutations in the predefined 23S rRNA and/or *parC* targets, identified in approximately 44.3% of *M. genitalium*-positive samples. This observation aligns with growing evidence of widespread resistance-associated mutations in global *M. genitalium* populations [13,19]. Notably, our data revealed that these mutations exhibited a non-random distribution within the predefined regions of the *M. genitalium* genome analyzed in this study, clustering into reproducible patterns, with few substitutions accounting for most detected variants. This structured distribution supports the suitability of targeted molecular assays for surveillance and their continued use in routine diagnostic workflows.

Among macrolide resistance-associated mutations, substitutions at positions A2059 and A2058 of the 23S rRNA gene (reported using *E. coli* numbering) were predominant. A2059G was the most frequently detected mutation, accounting for 52.8% of 23S rRNA mutation-positive samples, followed by A2058G (34.7%). These findings are consistent with those of previous surveillance studies from diverse geographic regions, which consistently revealed A2059G and A2058G as the predominant macrolide resistance-associated mutations in *M. genitalium* [14,33]. This concordance with the findings of previous reports supports the reliability of the molecular targets used in the multiplex PCR assays, underscoring these substitutions as key genetic markers of macrolide resistance.

The detection of dual substitutions at the same nucleotide position in some samples underscores the genetic complexity of *M. genitalium*. Although infrequent, these patterns suggest ongoing selective pressure and potential heterogeneity within bacterial populations [34,35]. Such patterns may indicate the presence of mixed *M. genitalium* populations within individual samples [36,37]. The clinical implications of these dual substitutions remain unclear; however, their detection supports the use of high-resolution molecular assays for identifying complex mutation patterns directly from clinical specimens.

Fluoroquinolone-associated *parC* mutations were detected in 1306 samples. G248T was the most prevalent substitution (52.2% of *parC* mutation-positive samples), followed by G259A (19.8%) and G248A (11.8%). This distribution is consistent with the findings of international studies reporting G248T (corresponding to S83I at the amino acid level) as the most common *parC* mutation associated with reduced fluoroquinolone susceptibility [13,14]. The recurrent detection of this substitution across populations suggests a conserved evolutionary pathway for fluoroquinolone resistance in *M. genitalium*.

Less frequent mutations, including G259C and G259T, were detected at low frequencies. The identification of samples harboring concurrent *parC* mutations further highlights the genetic heterogeneity of fluoroquinolone-associated resistance markers [5,16]. However, as with macrolide-associated mutations, these genetic alterations should be interpreted cautiously because phenotypic susceptibility testing and clinical outcome data were not used in this study.

Another key finding of our study is that 19.4% of *M. genitalium*-positive samples harbored concurrent mutations in both genes, indicating that a substantial proportion of samples may have reduced susceptibility to both major antimicrobial classes used in *M. genitalium* treatment.

The frequent co-occurrence of 23S rRNA and *parC* mutations observed in this study aligns with reports from other regions, where the increasing prevalence of dual-class resistance-associated mutations has raised concerns about narrowing treatment options [13,16]. Although genotypic findings do not necessarily translate into phenotypic resistance or treatment failure, these mutation patterns support the use of integrated molecular surveillance to monitor emerging resistance trends. Accordingly, the prevalence of resistance-associated mutations reported in this study should be interpreted cautiously and cannot be considered a direct indicator of clinical treatment failure at the individual patient level.

We also observed a higher prevalence of overall and concurrent mutations in male-associated samples than in female-associated samples. These differences persisted for 23S rRNA mutations and combined 23S rRNA-*parC* mutations but not for *parC*-only mutations. Similar sex-based differences have been reported, possibly reflecting differences in healthcare-seeking behavior and antimicrobial exposure, rather than biological sex differences [27,38,39].

In this study, urine specimens were more common among male-associated samples, whereas swab specimens were more common among female-associated samples. Thus, differences in specimen type may have contributed to the observed sex-associated variation in mutation prevalence [38,40,41], and specimen source may partially confound the interpretation of sex-based comparisons [42,43]. Rectal *M. genitalium* infection has been reported to be relatively common in certain populations, particularly among men who have sex with men [40]. Because anatomical site-specific data were not available in this laboratory-based dataset, it was not possible to determine whether rectal specimens were included. Consequently, variations in sampling practices may have influenced detection patterns independent of resistance-associated mutations.

In female-associated samples, the cervicovaginal microbiome may influence the persistence and detection of *M. genitalium* infection [44,45]. Alterations in the vaginal microbial community due to antimicrobial exposure not specifically targeting *M. genitalium* could indirectly affect infection dynamics [46]. Although microbiome data were not available in this study, this is an important area for future investigation. In addition to these biological considerations, characteristics of study population characteristics may also influence the interpretation of sex-associated differences [47]. The study population was predominantly composed of individuals aged 20–39 years; therefore, age-stratified analyses would have limited interpretability. However, future studies comprising more balanced age distributions could better evaluate potential age-specific resistance patterns.

These findings highlight the importance of presenting separate mutation distribution analyses separately for male- and female-associated samples, as sex-specific patterns may have implications for diagnostic strategies and resistance-guided treatment approaches. Interpretation of sex-associated differences should remain cautious given the potential influences of sampling practices and the absence of anatomical site-specific information. The lack of clinical treatment histories and outcome data also precludes the assessment of differential treatment responses. As such, the observed mutation patterns should be interpreted cautiously, and further studies incorporating standardized sampling strategies and detailed clinical data are warranted.

The primary strength of this study is its large sample size and the use of routine diagnostic data, minimizing selection bias and providing a realistic, representative overview of the contemporary molecular resistance landscape of *M. genitalium* in Korea. Furthermore, multiplex PCR assays enabled the simultaneous detection of *M. genitalium* and key resistance-associated mutations, supporting the integration of molecular surveillance into routine diagnostic workflows. This approach enabled the classification of samples into clinically relevant mutation pattern categories based on mutations in the 23S rRNA and *parC* genes.

Despite these strengths, some limitations should be acknowledged. First, our analysis was restricted to predefined mutation sites included in the multiplex PCR assays, and strain-level molecular typing (e.g., *mgpB* or *mgpC* sequencing) was not performed in this study; thus, rare or novel mutations outside the targeted regions and mixed-strain infections could not be assessed. Second, phenotypic susceptibility testing and clinical treatment outcome data were unavailable, which precluded direct correlation between detected mutations and clinical resistance. Third, the retrospective design and use of residual specimens limited access to detailed patient-level information, including antimicrobial exposure and symptom severity, and may have introduced potential selection bias. Fourth, the absence of anatomical site-specific information may have influenced the interpretation of sex-associated differences in mutation prevalence. Nevertheless, the consistency of our findings with previous surveillance studies supports the validity of our results and underscores the value of assay-based molecular surveillance as a complementary approach to traditional resistance monitoring.

## 5. Conclusions

This study provides a large-scale overview of macrolide- and fluoroquinolone-associated mutation patterns in *M. genitalium* detected through routine molecular diagnostics in Korea. Mutations in the predefined 23S rRNA and/or *parC* targets were detected in approximately 44% of *M. genitalium*-positive samples, with a notable proportion exhibiting concurrent mutations in both genes. The predominance of well-characterized substitutions, including A2059G and A2058G (23S rRNA) and G248T (*parC*), aligns with global surveillance data, supporting the ongoing use of targeted molecular assays.

Although genotypic findings do not directly infer phenotypic resistance, the generation of large-scale data on mutation distribution and co-occurrence obtained directly from routine diagnostic testing provides a practical resource for ongoing surveillance. Incorporating such assay-based approaches into routine clinical workflows may support resistance-guided management strategies and inform local treatment guidelines. However, future studies that involve integrating molecular data with phenotypic susceptibility testing and clinical outcomes are needed to elucidate the clinical utility of resistance-associated mutation surveillance in *M. genitalium*.

## Figures and Tables

**Table 1:** Demographic and specimen characteristics of *Mycoplasma genitalium*-positive samples.

Variable	Total (*n* = 4019)	Male (*n* = 1882)	Female (*n* = 2137)	*p*-Value
Age (years), median (IQR)	30 (25–38)	32 (27–40)	28 (23–34)	<0.001
Sample source, *n* (%)				<0.001
Urine	1923 (47.8)	1868 (99.3)	55 (2.6)	
Swab	2096 (52.2)	14 (0.7)	2082 (97.4)	

Continuous variables were compared using the Mann–Whitney U test and categorical variables were compared using Pearson’s chi-square test.

**Table 2:** Prevalence of 23S rRNA macrolide resistance-associated mutations stratified by sex.

Mutation	Total (*n* = 1253)	Male (*n* = 667)	Female (*n* = 586)
A2058C	5 (0.4%)	3 (0.4%)	2 (0.3%)
A2058G	435 (34.7%)	228 (34.2%)	207 (35.3%)
A2058T	142 (11.3%)	65 (9.7%)	77 (13.1%)
A2059C	54 (4.3%)	33 (4.9%)	21 (3.6%)
A2059G	661 (52.8%)	359 (53.8%)	302 (51.5%)
A2059T	0 (0%)	0 (0%)	0 (0%)

Percentages in the total column were calculated based on all 23S rRNA mutation-positive samples (*n* = 1253). Percentages in the Male and Female columns were calculated within sex-specific 23S rRNA mutation-positive samples.

**Table 3:** Prevalence of *parC* fluoroquinolone resistance-associated mutations stratified by sex.

Mutation	Total (*n* = 1306)	Male (*n* = 660)	Female (*n* = 646)
A247C	92 (7%)	40 (6.1%)	52 (8%)
G248A	154 (11.8%)	80 (12.1%)	74 (11.5%)
G248T	682 (52.2%)	380 (57.6%)	302 (46.7%)
G259A	259 (19.8%)	112 (17%)	147 (22.8%)
G259C	15 (1.1%)	6 (0.9%)	9 (1.4%)
G259T	108 (8.3%)	44 (6.7%)	64 (9.9%)

Percentages in the total column were calculated based on all *parC* mutation-positive samples (*n* = 1306). Percentages in the Male and Female columns were calculated within sex-specific *parC* mutation-positive samples.

**Table 4:** Distribution of combined mutation patterns stratified by sex.

Mutation Pattern	Total (*n* = 4019)	Male (*n* = 1882)	Female (*n* = 2137)	*p*-Value
No detected mutations	2240 (55.7%)	970 (51.5%)	1270 (59.4%)	<0.001
Any mutation	1779 (44.3%)	912 (48.5%)	867 (40.6%)	<0.001
23S rRNA only	473 (11.8%)	252 (13.4%)	221 (10.3%)	0.003
*parC* only	526 (13.1%)	245 (13%)	281 (13.1%)	0.906
23S rRNA + *parC*	780 (19.4%)	415 (22.1%)	365 (17.1%)	<0.001

Percentages were calculated based on the total number of analyzed *M. genitalium*-positive samples (*n* = 4019). *p*-values were calculated using Pearson’s chi-square test without continuity correction. Percentages in the Male and Female columns were calculated within each sex-specific group.

## Data Availability

The original contributions presented in this study are included in the article. Further inquiries can be directed to the corresponding author.

## Abbreviations

The following abbreviations are used in this manuscript:

*E. coli*	*Escherichia coli*
IRB	Institutional Review Board
*M. genitalium*	*Mycoplasma genitalium*
NAATs	Nucleic acid amplification tests
PCR	Polymerase chain reaction
rRNA	Ribosomal ribonucleic acid
